# Histology and RNA Sequencing Provide Insights Into Fusarium Head Blight Resistance in AAC Tenacious

**DOI:** 10.3389/fpls.2020.570418

**Published:** 2021-01-13

**Authors:** Kirby T. Nilsen, Sean Walkowiak, Santosh Kumar, Oscar I. Molina, Harpinder S. Randhawa, Raman Dhariwal, Brook Byrns, Curtis J. Pozniak, Maria A. Henriquez

**Affiliations:** ^1^Brandon Research and Development Centre, Agriculture and Agri-Food Canada, Brandon, MB, Canada; ^2^Grain Research Laboratory, Canadian Grain Commission, Winnipeg, MB, Canada; ^3^Morden Research and Development Centre, Agriculture and Agri-Food Canada, Morden, MB, Canada; ^4^Lethbridge Research and Development Centre, Agriculture and Agri-Food Canada, Lethbridge, AB, Canada; ^5^University of Saskatchewan, Saskatoon, SK, Canada; ^6^Crop Development Centre, College of Agriculture and Bioresources, University of Saskatchewan, Saskatoon, SK, Canada

**Keywords:** histology, QTL, breeding, resistance, AAC Tenacious, transcriptomics, FHB

## Abstract

Fusarium head blight (FHB) is a serious fungal disease affecting wheat and other cereals worldwide. This fungus causes severe yield and quality losses from a reduction in grain quality and contamination of grain with mycotoxins. Intensive breeding efforts led to the release of AAC Tenacious, which was the first spring wheat cultivar registered in Canada with a resistant (R) rating to FHB. To elucidate the physiological mechanisms of resistance, we performed histological and transcriptomic analyses of AAC Tenacious and a susceptible control Roblin after inoculation with *Fusarium graminearum* (*Fg*). The spikelet and rachis of infected wheat spikes were hand sectioned and monitored by confocal and fluorescent microscopy. Visible hyphae were observed within the inoculated spikelets for AAC Tenacious; however, the infection was largely restricted to the point of inoculation (POI), whereas the adjacent florets in Roblin were heavily infected. Significant cell wall thickening within the rachis node below the POI was evident in AAC Tenacious compared to Roblin in response to *Fg* inoculation. Rachis node and rachilla tissues from the POI and the rachis node below the POI were collected at 5 days post inoculation for RNAseq. Significant changes in gene expression were detected in both cultivars in response to infection. The rachis node below the POI in AAC Tenacious had fewer differentially expressed genes (DEGs) when compared to the uninoculated control, likely due to its increased disease resistance. Analysis of DEGs in Roblin and AAC Tenacious revealed the activation of genes and pathways in response to infection, including those putatively involved in cell wall modification and defense response.

## Introduction

Wheat (*Triticum aestivum* L.) is the most widely grown crop in Canada where it is sown to approximately 25 million acres (StatsCan, 2020). One of the greatest production constraints in wheat is disease pressure from fungi that cause Fusarium head blight (FHB), which is endemic throughout North America, Europe, and Asia. FHB is a disease complex caused by several different species within the *Fusarium* genus, with *F. graminearum* being the dominant species in North America (Trail, 2009). Damage due to FHB results in a reduction in both grain yield and quality. Trichothecene mycotoxins, such as deoxynivalenol (DON), are produced by the fungi and accumulate in cereal grains, which diminishes grain value, imposes export barriers, and poses serious health hazards to animal and human consumption (Jennings, 2007). Losses from FHB vary annually and depend on a multitude of factors, including moisture and humidity during anthesis, which is when the plants are most vulnerable to infection (Trail, 2009). The United States of America have reported losses from FHB to be in the billions of dollars, with significant losses occurring in the late 1990’s and early 2000’s (McMullen et al., 2012). In Canada, FHB has been reported to cause $1 billion in estimated losses in epidemic years, such as 2016 (Dawson, 2016). Disease incidence and severity for FHB has been increasing in North America over the past three decades, possibly due to agronomic practices (i.e., cereal-cereal crop rotations and no-till), climate change, and dynamics in the pathogen populations (Ward et al., 2008; Gilbert and Haber, 2013; Walkowiak et al., 2015). FHB has been difficult to manage because available fungicides do not provide complete FHB control; consequently, the most effective way to manage FHB is to use an integrated management strategy that includes the use of wheat cultivars with genetic resistance to the disease.

Genetic resistance to FHB is quantitative and is the result of several loci that contribute to molecular and physiological differences in the wheat plant. To date, more than 65 quantitative trait loci (QTL) have been identified that contribute to FHB resistance in wheat (Venske et al., 2019), including North American breeding material (McCartney et al., 2016) such as FL62R1 (Comeau et al., 2008; Zhang et al., 2018, 2020), as well as cultivars, such as Alsen, Glenn, Carberry, and AAC Tenacious (Bokore et al., 2017; Dhariwal et al., 2020). Pyramiding FHB resistance QTL increases disease resistance (Venske et al., 2019), but unfortunately, many FHB resistance QTL are also associated with poor agronomics and quality, making their transfer into elite varieties a challenge (Haile et al., 2019; Venske et al., 2019). By understanding the mechanisms and causal genes of resistance, breeders can more efficiently integrate resistance genes into elite material through next generation breeding technologies (i.e., transgenics) or targeted introgressions, thereby limiting the introduction of additional undesired genes and traits, which are often transferred through genetic linkage drag. Although most of the underlying genes that confer FHB resistance are currently either unknown or not validated on diverse germplasm, the availability of the wheat genome sequence is accelerating gene discovery and breeding efforts (IWGSC et al., 2018).

The Chinese cultivar Sumai 3 is one of the most studied FHB resistant wheat line thus far (Bokore et al., 2017). In Sumai 3, progression of the *Fusarium* mycelium through the parenchyma and vascular tissues of the rachis are impeded and less pervasive than in susceptible wheat cultivars, such as Roblin (Miller et al., 2004). *Fhb1*, which is derived from Sumai 3, is a major FHB resistance QTL and was recently cloned and controversially described to encode a chimeric lectin with agglutinin and pore-forming toxin-like domains (Rawat et al., 2016) and histidine-rich calcium-binding protein (Li et al., 2019; Su et al., 2019). A separate resistance gene derived from *Thinopyrum elongatum*, *Fhb7*, was identified to be a DON detoxification gene that encodes a glutathione S-transferase (Wang et al., 2020). Likewise, UDP-glycosyltranserases have been shown to detoxify DON and provide FHB resistance (Gatti et al., 2018). Molecular mechanisms of resistance are also likely to include pathogen recognition receptors (PRRs; Jones and Dangl, 2006), although the underlying genes are largely unknown. In addition to molecular mechanisms of resistance, physiological traits may also impact FHB resistance, including the presence of awns, heading date, anther extrusion, and plant height (Mesterházy, 1995; Klahr et al., 2007; Haile et al., 2019). Genes for some of these physiological traits in wheat have also been identified, such as the *Rht* genes for plant height (Würschum et al., 2017; Haile et al., 2019), though the mechanism of resistance to FHB may be indirect and is difficult to characterize (Haile et al., 2019; Venske et al., 2019). At a microscopic level, physiology within the wheat spike may also be important for disease resistance. For example, thickening of the cell walls may impact the spread of the fungus through the plant’s vasculature (Lahlali et al., 2016). Also, the rachis nodes may be a key point of defense against FHB, whereas DON contributes to the pathogen’s ability to overcome that defense (Jansen et al., 2005; Brown et al., 2010). Together, wheat breeders and researchers will benefit by more detailed dissection of FHB resistance mechanisms and their underlying genes.

Currently, the only FHB resistant spring wheat cultivar that is registered in Canada is AAC Tenacious; all other cultivars are either moderately resistant, intermediately resistant, moderately susceptible or susceptible (Brown et al., 2015). AAC Tenacious was developed by the Agriculture and Agri-Food Canada at the Cereal Research Centre in Winnipeg, Manitoba, and was registered in 2013. Recently, AAC Tenacious has been reported to possess major FHB resistance QTL using a bi-parental population AAC Innova × AAC Tenacious (Dhariwal et al., 2020). This study investigates the physiological and molecular characteristics of AAC Tenacious associated with resistance to FHB in the wheat spike.

## Materials and Methods

### Fungal Culture and Macroconidia Preparation

A highly virulent 3-acetyldeoxynivalenol producing isolate of *Fg* (HSW-15-39), obtained from the Henriquez Spring Wheat (HSW) collection of *Fusarium* isolates at Agriculture and Agri-Food Canada, Morden, Manitoba was used in this study. For spore production, approximately 1 cm^2^ of *Fg* preserved at −80°C (filter paper Whatman No.1) was placed in the center of a petri dish (100 mm) containing Spezieller-Nährstoffar Agar (SNA), and incubated at 22°C for 10 days under a combination of fluorescent-UV lights. Conidia suspensions were harvested in sterile water filtered through cheese cloth.

### Plant Materials

Plant materials used in this study included the spring wheat cultivars AAC Tenacious (Brown et al., 2015) and Roblin (Campbell and Czarnecki, 1987). AAC Tenacious is a high yielding spring wheat cultivar that belongs to the Canada Prairie Spring Red market class, derives from the cross HY667/BW346, and is resistant to FHB. Roblin is a spring wheat cultivar that is susceptible to FHB and derives from the cross RL4302/RL4356//RL4359 (Campbell and Czarnecki, 1987). AAC Tenacious is physiologically different from Roblin in being tall and resistant to FHB. Three seeds each of AAC Tenacious and Roblin were sown in 4” pots with a mixture of 50% Sunshine #5 soilless mix (Sungro, Horticulture Canada, Seba Beach, AB, Canada) and 50% soil, plus 6 *g* of Osmocote slow release fertilizer (14-14-14; Everris NA, Dublin Ohio, United States). Wheat plants were grown in controlled-environment cabinets with 16 h of light at 22°C and 8 h of dark at 15°C. Plant-prod 20-20-20 soluble fertilizer (Master Plant Products Brampton Ontario, Canada) was applied in the water at a rate of 0.75 g/L and 2 *g* of slow release fertilizer (14-14-14) was added 40 days after planting. At the three-leaf stage, the plants were thinned to one plant per pot. At mid-flowering stage (approximately 50% of anthers extruded), single floret inoculation was performed with water (control) or with 10 μL of *Fg* inoculum that was inserted between the lemma and palea at the midpoint of the spike using a micropipetter. For the *Fg* inoculation, the macroconidia suspension was prepared at a concentration of 5 × 10^4^ macroconidia/mL. Inoculated plants were covered with a plastic bag for 48 h to promote infection. This was repeated for six individual wheat plants for both AAC Tenacious and Roblin, and the number of spikelets demonstrating disease symptoms within each spike was recorded at regular intervals to assess type II FHB resistance.

### Tissue Processing and Confocal Laser Scanning Microscopy Analysis

**FIGURE 1:**
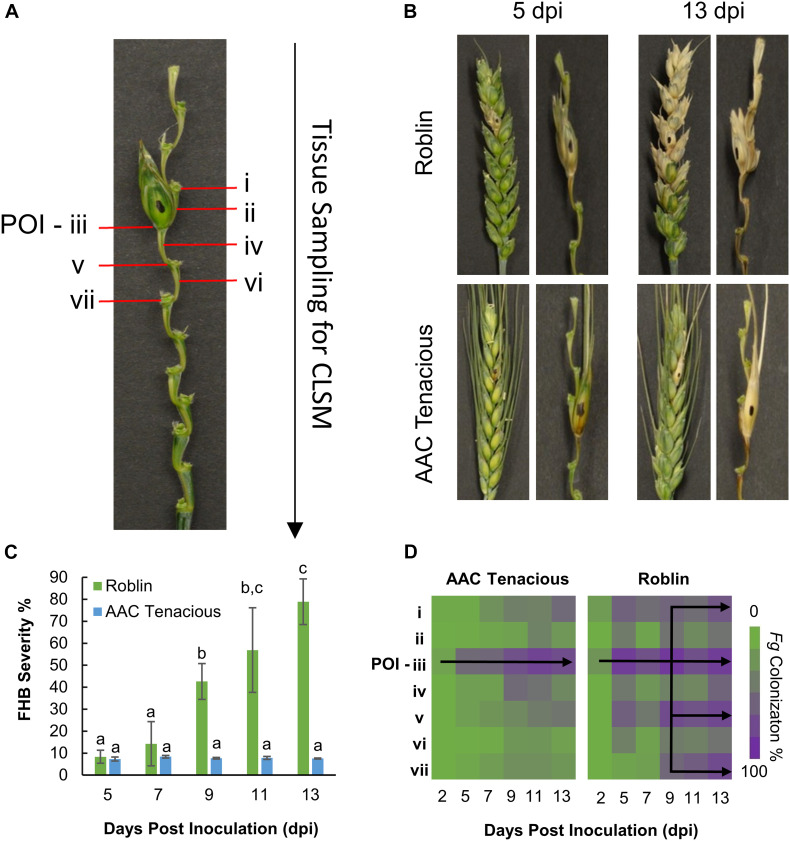
Disease assessment of AAC Tenacious and Roblin. **(A)** Tissue sampling for histological analysis by CLSM. **(B)** Wheat spikes at 5 dpi (left) and 13 dpi (right) inoculated with *Fg* for Roblin (top), and AAC Tenacious (bottom). For each time point, we show the intact wheat spike as well as a dissection that makes the rachis more visible. **(C)** FHB disease severity determined based on visual scoring of symptoms. Different letters above bars indicate statistically significant differences among treatments (*p* ≤ 0.05). **(D)** Percentage of colonization of *Fg* in the different spike sections (i–vii) in AAC Tenacious and Roblin from 2 dpi to 13 dpi, determined by CLSM.

In order to elucidate the course of *F. graminearum* infection in AAC Tenacious and Roblin, infection of wheat plants was performed as described previously and the spikelet and rachis of infected and control wheat spikes were hand sectioned at 2, 5, 7, 9, 11, and 13 days post inoculation (dpi). Tissue samples for each time point were hand sectioned while under magnification using the Zeiss Stemi 2000-C Stereo microscope. The samples were labeled sequentially from the top of the spike downward (Figure 1A). The rachis node (RN) and rachis internode (RI) immediately above the point of inoculation (POI) were designated “i” and “ii,” respectively. The POI was designated “iii.” The RI and RN below the POI were designated “iv” to “vii.” A minimum of three replications were collected for each of the seven positions on the spike (i–vii) for both cultivars across the different time-points. Samples were placed in 0.2 mL 8 strip-tubes and stained with Alexa Fluor^®^ 488 (Catalog No. W11261, Invitrogen^TM^; AF) and propidium iodide (Catalog No. P3566, Invitrogen^TM^; PI), following the manufacturer’s recommendations for the concentration of working solutions. The working solutions for AF and PI (10 μg/mL) were mixed to a 1:1 ratio, and 20 μL were added to each sectioned sample (i–vii). The strip-tubes were placed under vacuum for 20 min and protected from the light until microscopy analysis. Microscopy images were acquired using a Zeiss Laser Confocal LSM 700 laser scanning microscopy, with Zeiss Imager M2 microscope following excitation wavelength at 488 and 555 nm, by collecting the emitted fluorescence between 300–550 and 560–800 nm for AF and PI, respectively. Zeiss Efficient Navigation microscope software (ZEN) was used for image processing.

**FIGURE 2:**
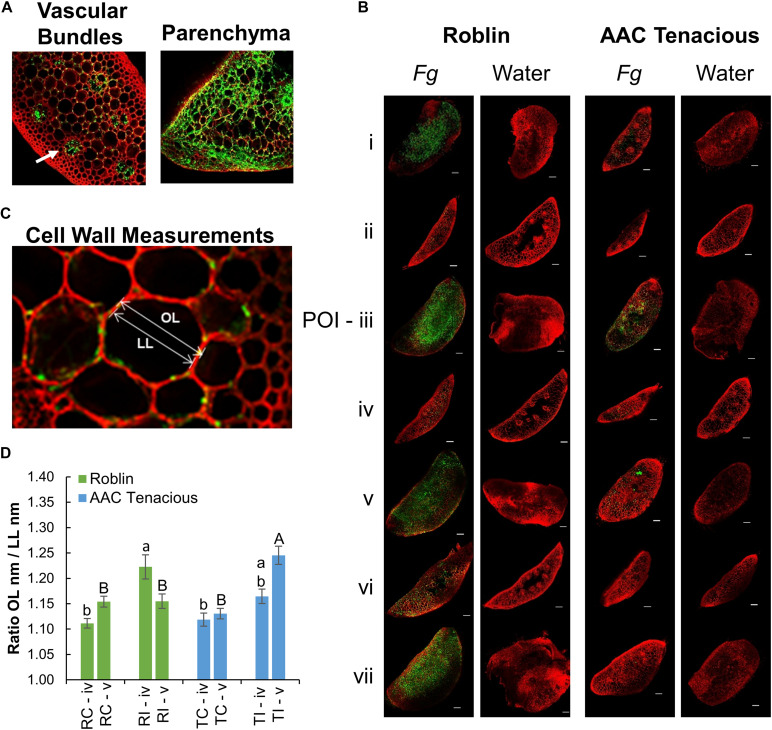
CLSM imaging of *Fg* infection. **(A)** CLSM images of cross sections showing the movement of the *Fg* through the vascular bundles (left), and infected vascular and parenchyma tissues (right) of Roblin. Infection within the vascular bundles is indicated (white arrow). Within the parenchyma, hyphae were observed both inter- and intracellularly (right). **(B)** Microscopic observation of the Fg infection process in the spike sections (i–vii) by CLSM analysis at 13 dpi. Fg and water inoculated tissue samples of Roblin (left) and AAC Tenacious (right) are shown. The scale bar represents 100 μm. **(C)** Measurement of the cell wall thickness was calculated using the ratio between the length of the outer periclinal cell wall (OL) and the length of the inner periclinal cell wall (LL). **(D)** Means of cell wall thickness across inoculated and non-inoculated cultivars within sections followed by different letters are significantly different, according to Tukey’s multiple comparison test (*p* ≤ 0.05). Error bars are ± 1 standard error of the mean.

Measurement of the cell wall thickness in the rachis internode (section “iv”) and rachis node (section “v”) was performed in parenchyma cells at 5 dpi modifying the protocol of Begović et al. (2015). The thickness of the cell walls was calculated using the ratio between the length of the outer periclinal cell wall (OL) and the length of the inner periclinal cell wall or cell lumen (LL) of two contiguous cells (Figure 2C). Measurements were done in nm using the Zeiss Efficient Navigation microscope software (ZEN). Cell wall thickness across treatments were analyzed in sections “iv” and “v” using the GLIMMIX procedure of SAS v.9.4 (SAS, 2014) with the effect of replicates as random. The effect of treatments (inoculated and non-inoculated cultivars) were considered as fixed. When a factor effect was significant, as indicated by significant *F* test (*p* ≤ 0.05), differences between the respective means were determined using the Tukey’s multiple comparison test (*p* ≤ 0.05).

Macroscopic disease symptoms of spikelets showing dark brown or bleaching symptoms and the proportion of infected spikelets in each spike (severity) was estimated for each time point. For quantification of the infection process at different time-points by Confocal Laser Scanning Microscopy (CLSM), the percentage of *Fg* colonization in the different sections of the spike were observed and recorded. Disease severity (%) was analyzed with GLIMMIX procedure of SAS v.9.4 (SAS, 2014) with the effects of replicates as random. The effect of time was considered as fixed. When a factor effect was significant, as indicated by significant *F* test (*p* ≤ 0.05), differences between the respective means were determine using the Tukey’s multiple comparison test (*p* ≤ 0.05).

### RNA Sequencing and Data Processing

Based on results from the CLSM analysis, at 5 dpi there were consistent phenotypic differences in the rachis node and rachilla between AAC Tenacious and Roblin; as such, we selected this time-point to examine gene expression patterns by RNAseq. The spikelet at the POI and the rachis node below the POI, sections “iii” and “v,” were isolated at 5 dpi for the *Fg* and water inoculations. One spike per plant/pot and 15 spikes were used per replicate. Each treatment was replicated three times. RNA extraction was performed from 360 sectioned samples (180 *Fg* inoculated and 180 water inoculated), using the Trizol Reagent (Ambion), according to the manufacturer’s instructions. All samples were treated with DNase I (Invitrogen), according to the manufacturer’s instructions. The RNA yield and quality were monitored using a Qubit 3.0 Fluorometer with the Qubit BR Assay Kit (Invitrogen), and an Agilent 2100 Bioanalyzer and the Agilent RNA 6000 Nano Kit (Agilent). The average RNA Integrity Number (RIN) value for all the samples was 9.0. cDNA libraries were prepared by using the NEB rRNA-depleted stranded (plant) kit. Samples were sequenced at the McGill University and Genome Quebec Innovation Centre (Montreal, Canada) using the HiSeq2500 Illumina sequencer with 125-nucleotide paired-end reads.

Analysis of the RNAseq reads was performed according to widely established standards and protocols for wheat (IWGSC et al., 2018). Briefly, adapters were trimmed from raw sequence reads using Trimmomatic, which were then aligned to the Chinese Spring RefSeq v1.0 wheat genome assembly (IWGSC et al., 2018) using STAR (Dobin et al., 2012). Using the available RefSeq v1.0 high-confidence gene annotations, a raw count matrix was generated for each annotated gene using HTSeq-Count (Anders et al., 2015) and imported into DESeq2 (Love et al., 2014) for differential expression analyses. Pairwise comparisons between treatments were considered, and genes were declared differentially expressed if the log_2_ fold change was greater than 2 or less than −2. Differentially expressed gene (DEG) lists were extracted for each comparison and analyzed for gene ontology (GO) enrichment in R using the topGO package (Rahnenführer, 2009). Variants were called for each sample using Freebayes^1^ software. Filtering and annotation of variants that differentiate AAC Tenacious from Roblin was performed using SnpSift and SnpEff software (Cingolani et al., 2012).

We then performed a more detailed inspection of variants within three QTL regions identified from Sumai 3 (*Fhb1*, *Fhb2*, and *Fhb5*). QTL regions were identified in RefSeqv1.0 based BLASTn analysis of markers *Gwm133* and *Gwm644* on chromosome arm 6BS for *Fhb2*, (Cuthbert et al., 2007), and *Gwm304* and *Gwm415* on chromosome arm 5AS for *Fhb5* (Xue et al., 2011). The *Fhb1* genomic region was identified using sequences of candidate genes and gene containing contigs [*Fhb1-1*, GenBank accession KU304333.1 (Paudel et al., 2020); *PFT* and *PFT* containing contig, GenBank accessions AY587018.1 and KX907434.1 (Rawat et al., 2016); and *TaHRC*, GenBank accession MK450312.1 (Su et al., 2019)].

## Results and Discussion

### Infection Patterns in AAC Tenacious and Roblin

Initially, we performed visual assessments of the *Fg* infection process in the wheat spikes, carefully observing fungal spread above and below the POI (Figure 1A). In the susceptible cultivar Roblin, there were no obvious visual symptoms of FHB infection at 2 dpi. At 5 dpi, Roblin consistently showed bleaching or dark brown lesions at the POI (section “iii”), which are typical symptoms of FHB infection (Figure 1B, top left). Symptoms began spreading outward to the adjacent spikelets until 13 dpi, ending in a final disease severity of 78.9% (Figure 1B, top right).

Similar to Roblin, disease symptoms were visible in AAC Tenacious at 5 dpi, as dark brown or bleaching symptoms at the POI (Figure 1B, bottom left). However, as time progressed, the spikelets above and below the POI had no observable disease symptoms, though brown lesions appeared on the rachis (Figure 1B, bottom right). At 9 dpi onward, the FHB severity in Roblin was significantly higher than that of AAC Tenacious (Figure 1C). The resistance we observed in AAC Tenacious is similar to Sumai 3, which is largely restricted the POI, albeit Sumai 3 has been reported to have visual symptoms above and below the inoculation site, whereas visual symptoms of AAC Tenacious are restricted to the POI (Ha et al., 2016; Wang et al., 2018).

To further characterize the infection spread in AAC Tenacious, we performed histological examination using confocal microscopy, which identified key differences in patterns of spread of *Fg* between Roblin and AAC Tenacious (Figures 1D, 2). Consistent with visual observations of disease symptoms, *Fg* mycelium was observed in the spikelets at 5 dpi for both cultivars (Figure 1D). The intracellular movement of the *Fg* occurred in both cultivars through the vascular bundles (Figure 2A, left), which is similar to what has been reported previously for BobWhite, Roblin, and Sumai 3 (Miller et al., 2004; Brown et al., 2010). Ultimately, both the vascular and parenchyma tissues were colonized in both cultivars at the POI; within the parenchyma, hyphae were observed both inter- and intracellularly (Figure 2A, right). Consistent with disease severity results (Figure 1C), the *Fg* colonization in Roblin was highest at POI and spread into the adjacent tissues by 13 dpi (Figure 1D, right, Figure 2B, left). In contrast, colonization of AAC Tenacious was mostly restricted to the POI, although there was sparse evidence of *Fg* in the tissues directly adjacent to the POI at 13 dpi (Figure 1D, left; Figure 2B, right). This suggests that although some infection may have spread beyond the POI in AAC Tenacious, it was highly restricted. A similar response was previously observed in Sumai 3, using a transformed *F. graminearum* strain. In that research, Sumai 3 displayed reduced *F. graminearum* spread through the parenchyma and vascular tissues of the rachis, when compared to Roblin (Miller et al., 2004). Our histological analyses supports previous reports indicating the importance of the rachis node as a barrier for disease resistance in wheat cultivars Nandu and BobWhite (Jansen et al., 2005; Ilgen et al., 2009; Brown et al., 2010), where the vascular tissues are hypothesized to become occluded in resistant wheat cultivars such as Sumai 3, thereby reducing fungal spread (Miller et al., 2004).

We performed a closer inspection of cell wall thicknesses in AAC Tenacious and Roblin at sections “iv” and “v” to identify differences that might be associated with FHB resistance (Figure 2C and Supplementary Figure 1). We did not observe significant differences in cell wall thicknesses in the control samples in AAC Tenacious and Roblin for section “iv” and “v,” nor were the cell walls significantly thicker between the cultivars in section “iv” after inoculation with *Fg* (Figure 2D and Supplementary Figure 1). Our finding is different to what was reported previously for Sumai 3, where cell wall thickening was identified in the surrounding vascular bundles in the inoculated rachis of the susceptible cultivar Muchmore (Lahlali et al., 2016), but thickening was less prevalent in the resistant cultivar Sumai 3. However, we did observe differences in cell wall thicknesses of the infected tissues in section “v” after *Fg* inoculation. The cell walls in the rachis node (section “v”) were thicker in AAC Tenacious than in Roblin in response to *Fg* infection (Figure 2D). This finding supports the hypothesis that occlusion at the rachis node below the POI may be a mechanism of FHB resistance in AAC Tenacious. Miller et al. (2004) proposed that early occlusion of the vascular bundles in the rachis node of Sumai 3 plays a role restricting the spread of *F. graminearum* in the spike and it is a component of type II resistance. It has been reported that inhibition of trichothecene synthesis in *F. graminearum* causes the fungus to become blocked by the development of heavy cell wall thickenings in the rachis node in the wheat cultivar Nandu (Jansen et al., 2005). The previous described relationships between cell wall thickening, defense at the rachis node, and the inhibition of DON is intriguing, and warrants further investigation as a possible mechanism of resistance in AAC Tenacious. In summary, our findings indicate that colonization occurs via the vascular and parenchyma tissues and that the spread of infection was slower and less widespread in the rachis and adjacent spikelets in AAC Tenacious when compared to Roblin, possibly due to cell well thickening at the rachis node.

### Global Trends in Gene Expression During Fusarium Infection of AAC Tenacious and Roblin

Dynamics in gene expression during pathogen infection have been useful to identify genes and pathways involved in defense response. Recently, gene expression analysis was able to delineate genes responsive to FHB infection in four wheat genotypes (Pan et al., 2018), as well as implicate candidate genes involved in FHB resistance in wheat lines derived from Sumai 3 (Gadaleta et al., 2019). We performed RNAseq of Roblin and AAC Tenacious at the POI (section “iii”) and the rachis node below the POI (section “v”) at 5 dpi (Figure 1A) to further investigate genes responsive to FHB infection in these two tissues, particularly, in section “v” where we observed phenotypic differences in FHB symptoms and cell wall thickening in AAC Tenacious (Figure 2D). A complete summary of differential expression analysis is presented in Supplementary Dataset 1.

**FIGURE 3:**
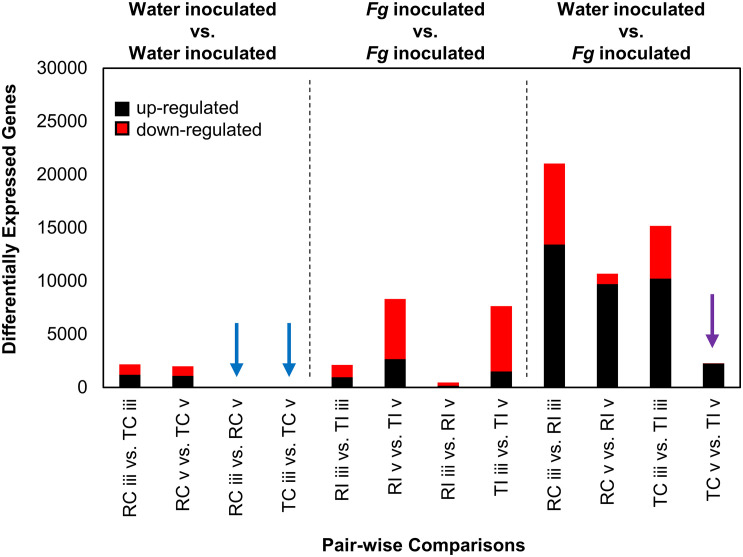
DEGs from *Fg* and water inoculated samples of AAC Tenacious and Roblin. The number of DEGs (both up- and down-regulated) in each pair-wise comparison are shown. Conditions are abbreviated to two letter labels where the first letter is the cultivar (Roblin is “R” and AAC Tenacious is “T”), the second letter indicates the inoculum (*Fg* inoculated is “I” and the water control is “C”), which is followed by the tissue section (POI is “iii” and adjacent spikelet is “v”). Comparisons between water control samples are indicated (left), with comparisons with no differentially expressed genes indicated by blue arrows. Comparisons between *Fg* inoculated conditions are also shown (middle). Comparisons between *Fg* and water inoculations are indicated (right), where purple indicates comparisons between AAC Tenacious *Fg* inoculated and water controls samples at section “v.” Depending on the comparison, up-regulated genes are considered to have increased expression in the *Fg* inoculated sample, AAC Tenacious, or “section v”.

Remarkable transcriptional stability was observed in intracultivar comparisons between sections “iii” and “v” in the water inoculated controls, as no DEGs (*n* = 0) were identified between the two tissue types in these comparisons (Figure 3, blue arrows). This indicated that the two tissues are functionally similar in the absence of FHB. We observed ∼2,000 DEGs in the intercultivar comparisons of the water inoculated controls at sections “iii” and “v,” indicating that there are some differences in gene expression or read mapping biases due to allelic variation between cultivars (Figure 3). As expected, gene expression patterns were vastly different in the comparisons between AAC Tenacious inoculated at section “v” and all other inoculated conditions (Figure 3). In contrast, inoculated AAC Tenacious at section “v” was more similar to the water inoculated controls, with ∼2,300 DEGs in intracultivar comparisons (Figure 3, purple arrow). These patterns are consistent with our observation that disease symptoms and colonization in AAC Tenacious was mostly restricted to the POI, whereas infection had spread to adjacent tissues in Roblin. The greatest number of DEGs, ∼20,000, were observed in the intracultivar comparisons of the *Fg* treated and the water inoculated controls at section “iii” (Figure 3), indicating a significant change in the transcriptional landscape in response to *Fg* infection. A similarly large response in DEGs was also observed in a separate RNAseq study involving *Fg* infection of four wheat genotypes; albeit, the inoculum and experimental design are different than this study (Pan et al., 2018). There were notably few DEGs, ∼2,100, in the intercultivar comparison at section “iii” (Figure 3), suggesting that the response to *Fusarium* is similar at the point of infection between the two cultivars. Although it is possible that some of these genes could contribute to a differential response to *Fg*, giving rise to resistance in AAC Tenacious, or susceptibility in Roblin.

Genes that were differentially expressed between cultivars at sections “iii” and “v” during *Fg* infection showed enrichment in categories including defense response (GO:0042742, GO:0006952, GO:0098542, and GO:0050832), response/defense to fungus (GO:0009620, GO:0050832), cell wall organization (GO:0071555), and response to stress/oxidative stress (GO:0006979, GO:0006950). These functions are consistent with genes involved in defense response or cell wall thickening in response to *Fg* infection and suggest a differential response to *Fg* at the POI and rachis node below the POI by AAC Tenacious. A complete set of enriched GO terms from all pair-wise comparisons is provided in Supplementary Dataset 2.

### Gene Level Expression Patterns Provide Insights Into Possible Mechanisms of Resistance in AAC Tenacious

Given the observed differences at the rachis node between Roblin and AAC Tenacious, we performed a more detailed inspection of the expression differences in individual genes within this region (section “v”) in infected tissues. By filtering for genes that were differentially expressed (*p* < 0.01 and log_2_ FC < −2, > 2) between inoculated Roblin and AAC Tenacious at section “v,” and not differentially expressed in intercultivar comparisons with the water inoculated controls, we generated a list of 2,518 genes that may be involved in the differential response to *Fg* (Supplementary Dataset 3). Given that we identified cell wall thickening in AAC Tenacious within the rachis node, it is plausible that differential expression of genes involved in cell wall modification identified in our analysis are contributing to resistance to *Fg*. Top DEGs based on fold change between Roblin and AAC Tenacious at section “v” are summarized in Table 1. Among these, we identified a number of cell wall genes putatively encoding proteins such as endoglucanase, expansin protein, methylesterase, laccase, and various transcription factors. Other genes encoding cell wall related proteins, such as cellulose synthase (*TraesCS5D01G261000, TraesCS6A01G 169200, TraesCS6B01G197200, TraesCS7A01G331500*, and *TraesCS7B01G290000*), xyloglucan endotransglucosylase/hydrolase (*TraesCS2D01G484000*), and several beta glucosidase genes were also differentially expressed in our list, but were not among the top DEGs (Supplementary Dataset 3). Four genes encoding putative proteins with similarity to glutathione S-transferase were also identified to be differentially expressed between Roblin and AAC Tenacious (Table 1), similarly *Fhb7* was recently identified to be a DON detoxification gene that encodes a glutathione S-transferase (Wang et al., 2020).

**TABLE 1:** Top 20 up-regulated, and 20 down-regulated DEGs in the intercultivar comparison between inoculated AAC Tenacious (TI) and Roblin (RI) at the rachis node below the POI (section “v”).

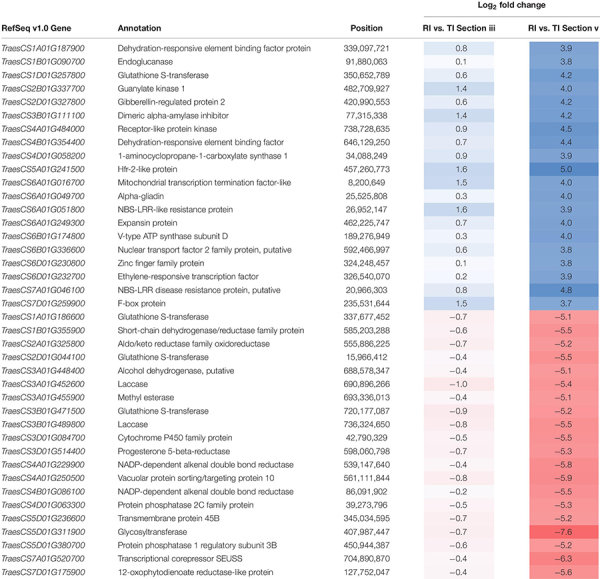

*The RefSeq v1.0 gene name is shown along with annotation and the position on the chromosome. Shaded values on the right of the table are log_2_ fold change ratio of expression values, where blue indicates upregulation, and red indicates down-regulation in AAC Tenacious. This list is a subset a complete list containing 2,518 genes that were identified for this comparison. The full list is presented in Supplementary Dataset 3.*

Closer inspection of the genes that are differentially expressed across all comparisons identified two genes located on the long arms of chromosomes 6B and 5A, *TraesCS5A01G236200* (phosphatidylinositol-4-phosphate 5-kinase family protein) and *TraesCS6B01G441400* (leucine-rich repeat receptor-like protein kinase family protein), that were consistently differentially expressed between Roblin and AAC Tenacious, both in control and *Fg* inoculated samples (Supplementary Dataset 1). These two genes were also reported to be differentially expressed between the susceptible cultivar Shaw, and three resistant wheat genotypes Nyubai, Wuhan 1, and HC374 (Pan et al., 2018). Another gene, *TraesCS7B01G415600* (protein kinase), was also highly differentially expressed in inoculated tissues between Roblin and AAC Tenacious, and was differentially expressed between Shaw and resistant genotypes (Pan et al., 2018). Additional research on these genes may support a role in FHB response or defense.

### Sequence Diversity and Expression of Genes in Genomic Regions

In addition to analyzing genome wide trends in gene expression, we performed variant analysis using our transcriptome data to identify candidate regions of the genome that may be different between Roblin and AAC Tenacious. Analysis of three QTL regions identified from Sumai 3 (*Fhb1*, *Fhb2*, and *Fhb5*) indicated that they were located at 3,309,706–8,801,036 on chromosome 3BS (*Fhb1*), 227,282,705–313,887,994 on chromosome arm 6BS (*Fhb2*), and 105,433,775–214,166,300 on chromosome arm 5AS (*Fhb5*). We detected clusters of variants near *Fhb1* and *Fhb2* genomic regions on chromosome arms 3BS and 6BS, respectively, but fewer variants near *Fhb5* on chromosome arm 5AS (Supplementary Figure 2 and Supplementary Dataset 4). Additional research is needed to determine if these variants may be associated with differences in FHB response between Roblin and AAC Tenacious. Nevertheless, these regions contained fewer variants than many of the other regions of the genome (Supplementary Figure 2). Curiously, chromosomes 5A and 6B contained the greatest rate of sequence variants (>4 variants per Mb); however, these were largely located on the long arms of the chromosomes (Table 2, Supplementary Figure 2, and Supplementary Dataset 4).

**TABLE 2:** Summary of variants identified on each chromosome that differentiate AAC Tenacious from Roblin.

**Chromosome**	**Length**	**Variants**	**Variants/Mb**
1A	594,102,056	1,430	2.41
1B	689,851,870	2,101	3.05
1D	495,453,186	457	0.92
2A	780,798,557	1,466	1.88
2B	801,256,715	3,085	3.85
2D	651,852,609	820	1.26
3A	750,843,639	1,299	1.73
3B	830,829,764	2,338	2.81
3D	615,552,423	273	0.44
4A	744,588,157	606	0.81
4B	673,617,499	1,572	2.33
4D	509,857,067	210	0.41
5A	709,773,743	2,861	4.03
5B	713,149,757	2,267	3.18
5D	566,080,677	488	0.86
6A	618,079,260	1,961	3.17
6B	720,988,478	3,076	4.27
6D	473,592,718	660	1.39
7A	736,706,236	1,288	1.75
7B	750,620,385	1,548	2.06
7D	638,686,055	483	0.76
Un	480,980,714	359	0.75

*A full list of variants is presented in Supplementary Dataset 4.*

**FIGURE 4:**
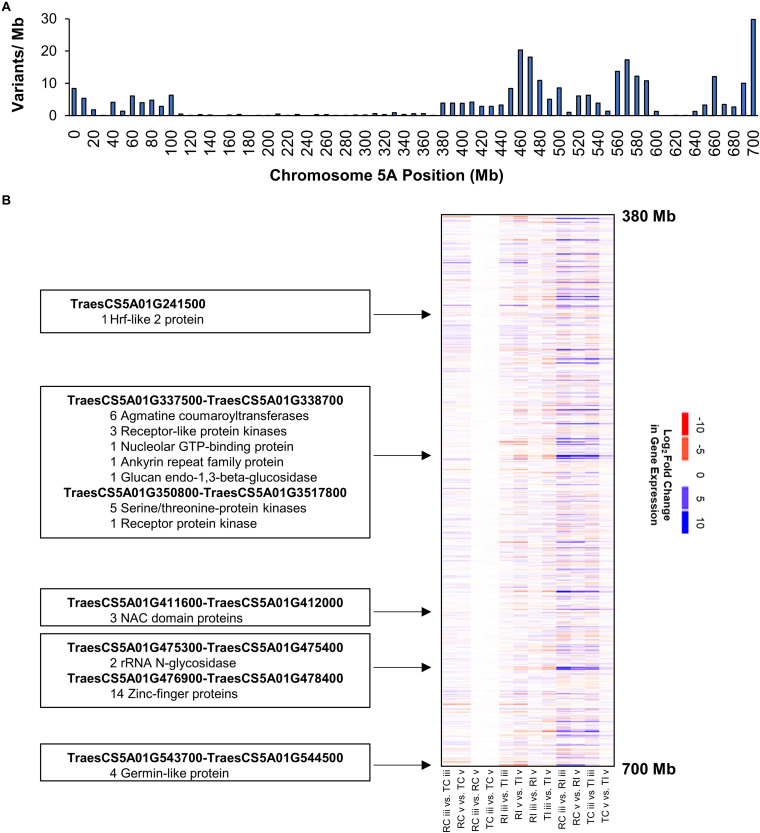
Chromosome 5AL has high nucleotide diversity between AAC Tenacious and Roblin and carries genes responsive to *Fg* infection. **(A)** Barplot of variants along chromosome 5A filtered to differentiate AAC Tenacious from Roblin. **(B)** Heatmap of gene expression within the interval containing FHB responsive genes. A full summary of differential expression analysis is presented in Supplementary Dataset 1. The colors are scaled according to a log_2_ fold change in gene expression for each possible pairwise comparison between treatments. Conditions are abbreviated to two letter labels where the first letter is the cultivar (Roblin is “R” and AAC Tenacious is “T”), the second letter indicates the inoculum (*Fg* inoculated is “I” and the water control is “C”), which is followed by the tissue section (POI is “iii” and rachis node is “v”). Clusters of differentially expressed genes and their putative functions are indicated.

Variants on the long arm of chromosome 5A clustered into three distinct peaks located at positions 460, 570, and 700 Mb (Figure 4A). This pattern suggests chromosome 5AL is a region of nucleotide sequence divergence between AAC Tenacious and Roblin, as such we performed a more detailed investigation of DEGs in this region to serve as an example. We detected several clusters of genes showing strong differential expression in response to inoculation with *Fg* on chromosome 5AL (Figure 4B). These included several multi-gene clusters composed of members of the agmatine coumaryltransferase gene family that have been previously implicated in FHB resistance (Kage et al., 2017). Agmatine is a potent inducer of DON production by *Fg* (Gardiner et al., 2009), which is required for the spread of infection of wheat (Bai et al., 2002; Jansen et al., 2005). Receptor like kinases and serine/threonine kinases were also differentially expressed in response to *Fg* within the chromosome 5AL interval. These genes can recognize pathogen associated molecular patterns and signal for the activation of defense response pathways (Jones and Dangl, 2006; Sari et al., 2019). Other FHB responsive gene clusters on 5AL included NAC transcription factors, rRNA N-glycosidases, zinc-finger proteins, and germin like proteins (Figure 4B). Recently, the major FHB reylsistance gene *Fhb1* was described to encode a chimeric lectin with agglutinin and pore-forming toxin-like domains (Rawat et al., 2016), and a gene, encoding a pore forming toxin-like protein *Hfr-2* (*TraesCS5A01G241500*), was also identified on chromosome 5AL that was differentially expressed between inoculated AAC Tenacious and Roblin at section “v” (Figure 4B). Further research is needed to confirm the association between genomic regions harboring candidate genes and the resistance in AAC Tenacious.

## Conclusion

To our knowledge, this is the first physiological and RNAseq analysis of AAC Tenacious under *F. graminearum* infection. Through macro and microscopic analysis of replicated infection studies, we were able to determine that AAC Tenacious can restrict the spread of *Fg* within infected wheat spikes, with visual symptoms mostly restricted to the POI. Our findings suggest that cell well thickening at the rachis node may play a role in restricting *Fg* spread in AAC Tenacious. Analysis of DEGs between Roblin and AAC Tenacious and GO analysis identified FHB responsive genes involved in defense response, response to fungi and stress, cell wall organization, and were most associated with the cellular membrane. Using variant analysis, we identified chromosomes with high nucleotide diversity such as on 5AL that differentiated Roblin and AAC Tenacious. These variants will be useful for the development of improved molecular markers that can assist in cloning of causal genes that are involved in FHB resistance. In addition, we identified genes on chromosome 5AL that could be affecting fungal pathways involved in infection and/or the production of DON. Our results suggest the FHB resistance in AAC Tenacious may be attributed to thickening of the cell walls within the rachis node, combined with activation of defense response pathways. Functional genetics and fine mapping studies involving large genetic mapping populations are needed to validate the involvement of these and other regions involved in the FHB response and could identify causal markers for the type II resistance in AAC Tenacious.

## Data Availability Statement

Transcriptome read data presented in this article has been deposited in the Sequence Read Archive (https://www.ncbi.nlm.nih.gov/sra) BioProject ID: PRJNA630776.

## Author Contributions

KN and SW performed bioinformatics analyses on RNA-Seq dataset, prepared tables and figures, writing – original draft, and writing – review and editing. MH conceived and designed the experiments, funding acquisition, project administration, performed plant inoculations, sample processing and RNA extractions, generated the data, analyzed the CLMS photos, prepared tables and figures, writing – original draft, and writing – review and editing. OM performed the statistical analysis and writing – review and editing. SK, HR, RD, BB, and CP contributed to writing – review and editing. BB and CP provided computational resources. All authors read and approved the final manuscript.

## Conflict of Interest

The authors declare that the research was conducted in the absence of any commercial or financial relationships that could be construed as a potential conflict of interest.

## Footnotes

1 https://github.com/ekg/freebayes

## Supplementary Material

Supplementary Figure 1 CLMS images of cross sections demonstrating cell wall thickening in response to *Fg*. Water (left) and *Fg* (right) treatment of tissues from section “iv” and “v” are shown for AAC Tenacious (top) and Roblin (bottom) at 5 dpi.

Supplementary Figure 2 Genome wide analysis of variant density that differentiate AAC Tenacious from Roblin. The position along the *x*-axis is in Mb.

Supplementary Dataset 1 Genome wide differential gene expression results for each pairwise treatment comparison in the transcriptomic analysis. Table values are in log_2_ fold change for each comparison.

Supplementary Dataset 2 GO enrichment *p-*value matrix for each pairwise treatment combination in the transcriptomic analysis.

Supplementary Dataset 3 Candidate differentially expressed genes between Roblin and AAC Tenacious at the rachis node below the POI (section “v”). Filters were applied requiring no differential expression in intercultivar comparisons between water inoculated controls, and differential expression in the comparison RI vs TI at section “v”.

Supplementary Dataset 4 Annotation of filtered variants and their predicted functional effects that differentiate AAC Tenacious from Roblin. Variant calls are highlighted based on their scoring with reference (green) and variant (red).
