# Supplementary figures and images for: Histology and RNA Sequencing Provide Insights Into Fusarium Head Blight Resistance in AAC Tenacious
Source: Front Plant Sci. 2021 Jan 13;11:570418. doi: 10.3389/fpls.2020.570418 (PMC7838103; doi:10.3389/fpls.2020.570418)

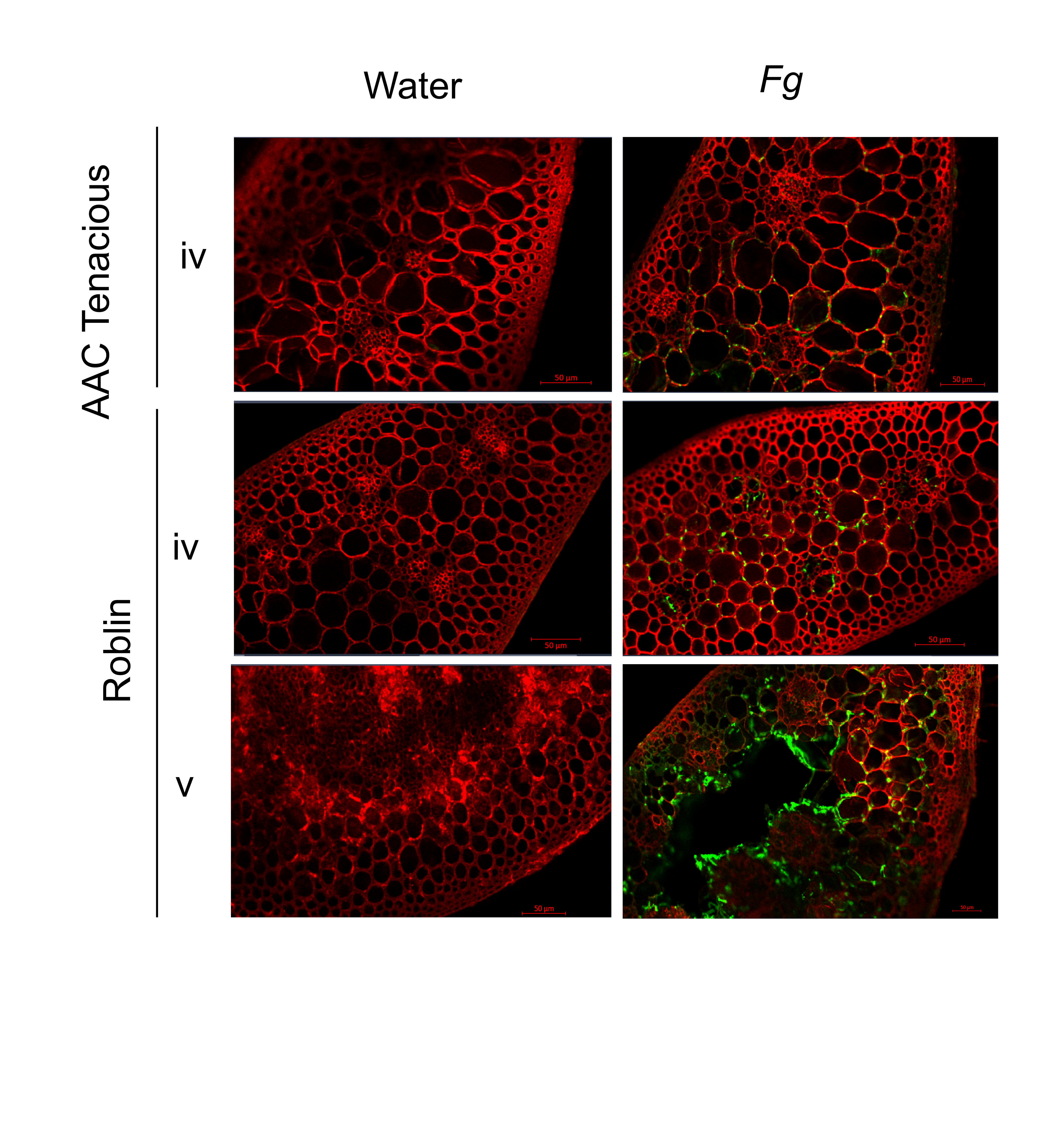

Supplement: Supplementary Figure 1 — CLMS images of cross sections demonstrating cell wall thickening in response to Fg. Water (left) and Fg (right) treatment of tissues from section “iv” and “v” are shown for AAC Tenacious (top) and Roblin (bottom) at 5 dpi. [file Image_1.TIF]

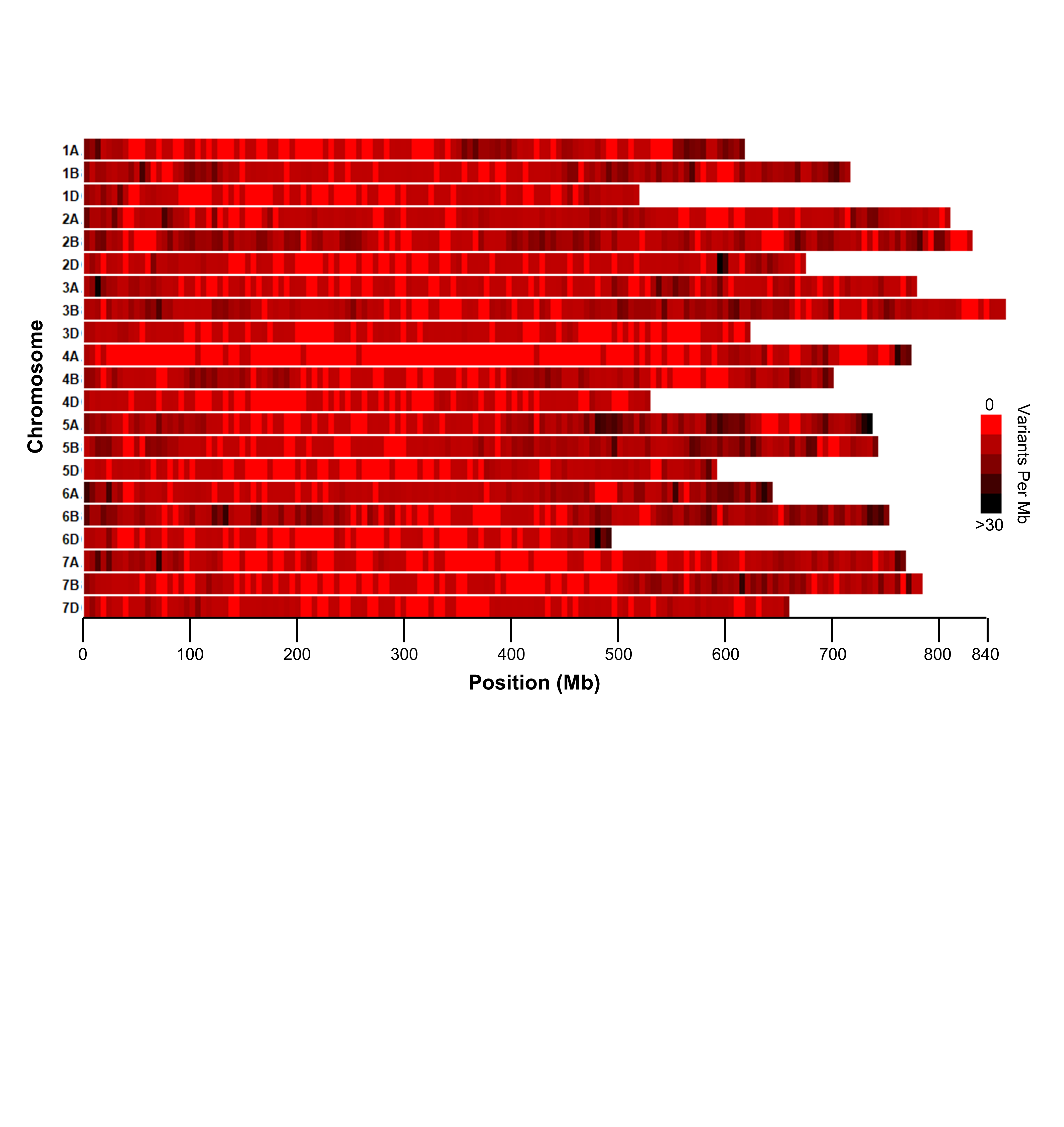

Supplement: Supplementary Figure 2 — Genome wide analysis of variant density that differentiate AAC Tenacious from Roblin. The position along the x-axis is in Mb. [file Image_2.TIF]
